# Cerebral Affections Often Mistaken for Neoplasm

**Published:** 1911-12

**Authors:** Tom A. Williams

**Affiliations:** MB. CM., (Edin.); Washington, D. C.; Memb. Corresp. Socs de Neurol. and Psychol de Paris; Memb. Assoc. Med. Ment. Clin. etc.; Neurologist to Epiphany Free Dispensary


### CEREBRAL AFFECTIONS OFTEN MISTAKEN FOR NEOPLASM. DIFFERENTIAL DIAGNOSIS OF ACTUAL CASES.

By Tom A. Williams, MB. CM., (Edin.), Washington, D. C

*Memb. Corresp. Socs de Neurol. and Psychol de Paris; Memb. Assoc. Med. Ment. Clin. etc.; Neurologist to Epiphany Free Dispensary.*

It will be instructive to relate a few examples which presented symptoms which aroused suspicion of a neoplasm in or near the cerebrum, which in some cases were derived from other cases than neoplastic growth.

If Cushing's experience is the usual one, the commonest mistake where a tumor affects the cerebrum is to diagnose hysteria. Formerly there was no excuse for this, as hysteria was supposed to be characterized by certain stigmata, the presence of which was supposed to be enough for a diagnosis. But we know now that such a stigma as inverted color fields is a sign not of hysteria, but of increased intracranial tension. We know also that hysterical anaesthesia is induced, evanescent and easily removed. We know that the hysterogenetic zones are merely the normal erethistic areas which most easily provoke instinctive efense reactions, and the intensity of these varies in different individuals without necessary relation to their hysterizability, or that they may be the artifacts of suggestion. Contractures and palsies, too, are now also clearly comprehended as direct or indirect results of suggestion from without or within. Without further citation, we can, in short, affirm that the stigmata are just as psychogenic and as variable by suggestion as are the accidents of hysteria themselves; for it is now shown that most of the trophic disorders which psychological mechanisms are incapable of produing are either the results of intention, whether from mythomanic disorder or the result of deliberate simulation, whether puerile or ingenious; or else that they are the signs of such disorder as trophoedema, vascular or secretory neurosis; in short, that they are non-psychogenic and have nothing to do with hysteria.

Again, the deep reflexes are not in reality modified hysterically, although here two qualifications must be mentioned.

First, by imitative suggestion, a reflex may appear to be modified, but it is not difficult as a rule to detect intentional interference of the patient with the response of his lower neurones. A very little address and experience suffices to detect this.

Secondly, Systemic states which interfere with the nutrition of the neurones often modify reflex responses, and sometimes do so unequally in different parts of the body. Such toxic states very often also increase the patient's suggestibility by lowering the threshold of the associational processes. In this way is presented a picture of somatic perturbed reflectivity along with hypersuggestibility. Such a picture is familiar to all of us in cases of incipient paresis, severe alcoholism and in many acute toxi-infectious states. It is not legitimate to say that the hysterical state of these patients is responsible for the perturbation of their reflexes; for a common pathogene is the source of both.

When these considerations are borne in mind, and they are applied with a knowledge of the technique of clinical neurology, very many fewer cases of cerebral neoplasm will be labelled hysteria, for the presence of any of the preceding non-psychogenic signs will no longer be accepted as hysterical, but will arouse the ertainty of somatic disorder.

A pregnant illustration of these considerations was the case of a young girl who was one summer in the Salle Pinel at the Salpetriere and whom Professor Dejerine treated for several months as a hystero-neurasthenic on account of intellectual torpor, peculiarity of disposition, of language, of humor, and fugitive headaches. The following winter I observed the same patient at the Hotel Dieu, where she was admitted to Professor Ballet's ward because of an amnestic aphasia for substantives. It was not until I detected an incipient papilloedema some weeks later that neoplasm was diagnosed.

The preceding case is merely an illustration of how even great neurologists blundered before the ideas of Babinski regarding hysteria had prevailed. The following case illustrates the procedure now that those ideas can be utilized.

1. There follow cases of hysterical monoplegia in the presence of an actual neoplasm.

2. Luetic meningitis of the anterior torsa with uncinate gyrus syndrome.

3. Intermitten claudication of cerebral vessel causing periodic hemiplegia.

4. Cerebral oedema from nephritis.

But delicate though the diagnoss may be when the symptoms of cerebral tumor are presented in cases of luetic meningitis, intermittent claudication of cerebral vessels or when a clinical picture of hysteria is presented in a syndrome created by a neoplasm, the diagnosis is perhaps more difficult still when we are dealing with results of nephrites. As the functional efficiency of the kidneys undergoes variations as single measurement of its excretory, even by phenol-suphonphthalein capacity even may not be sufficient for a diagnosis; and a slight quantity of albumin in the urine by no means precludes the possibility of a cerebral neoplasm in addition to a nephritis. The papilloedema in neither case has pathoglominic characters as was formerly believed.

Uremic oadema may occur focally and give rise to inequalities in feflectivity or may preponderate upon certain portions of the cerebrum, causing a predominance of symptoms in either disordered motility, sensibility, equilioration or intelligence. Indeed, arterio-schlerotic changes may lead to disorganization of some region and produce permannte symptoms still more suggestive of cerebral invasion.

But the most difficult diagnosis of all is when we have to deal with cases of so-called serous meningitis, for the relief these cases obtain from accropuncture of the ventricle may not be permanent and is of the same character as is that gained when the stasis of fluid due to neoplastic abstraction. As we are not yet in possession of sufficiently precise critera for dagnosing this kind of what has been called pseudo tumor.

